# Late recurrence of breast carcinoma metastasis to the hypopharynx: a case report

**DOI:** 10.1186/s40064-016-2226-1

**Published:** 2016-05-11

**Authors:** Chisako Izumi, Kiyoshi Misawa, Shiori Endo, Kenichi Sugiyama, Daiki Mochizuki, Atsushi Imai, Masato Mima, Yuki Misawa, Takashi Yamatodani, Hiroyuki Mineta

**Affiliations:** Department of Otolaryngology/Head and Neck Surgery, Hamamatsu University School of Medicine, 1-20-1 Handayama, Shizuoka, 431-3192 Japan

**Keywords:** Hypopharynx, Breast carcinoma metastasis, E-cadherin, VEGFR2, Aromatase inhibitor

## Abstract

**Background:**

We report a rare case of a patient with a hypopharyngeal metastasis from breast cancer.

**Case presentation:**

Isolated breast cancer metastasis to the hypopharynx has been previously reported in only one autopsy case. Herein, we report a 56-year-old woman with metastases to the hypopharynx almost 24 years after receiving a mastectomy and chemotherapy to treat primary breast carcinoma. We believe that she is the first patient to be treated for metastatic breast carcinoma to the hypopharynx. The hypopharyngeal tumor reduced in size after administration of an oral aromatase inhibitor. The patient has remained alive with a preserved larynx for three years.

**Conclusions:**

Breast cancer metastasis to the hypopharynx is an extremely rare event.

## Background

In women, breast cancer is diagnosed in approximately 23 % of all cancer cases, which is the highest proportion amongst all cancer types and accounts for approximately 7.6 million deaths worldwide each year. Approximately 90 % of these deaths are due to metastatic dissemination of the disease (Cummings et al. 2014). In one study, the most common sites of the first metastasis of breast cancer were the bone (35 %), lungs (23 %), skin (22 %), and regional lymph nodes (16 %) during an average observation period of 3.6 years (range 0.8–6.4 years) (Kamby et al. 1988). The incidence of metastatic carcinoma in the hypopharynx is extremely low. To our knowledge, only one autopsy case has been previously reported (Nguyen and Weitzner 1983).

Here, we report a case of dormant breast carcinoma metastasis to the hypopharynx. We also evaluated E-cadherin and VEGFR2 expression levels and methylation statuses in order to help elucidate the mechanism of dormant ER-positive breast cancer metastasis in this case.

## Case presentation

**Fig. 1:**
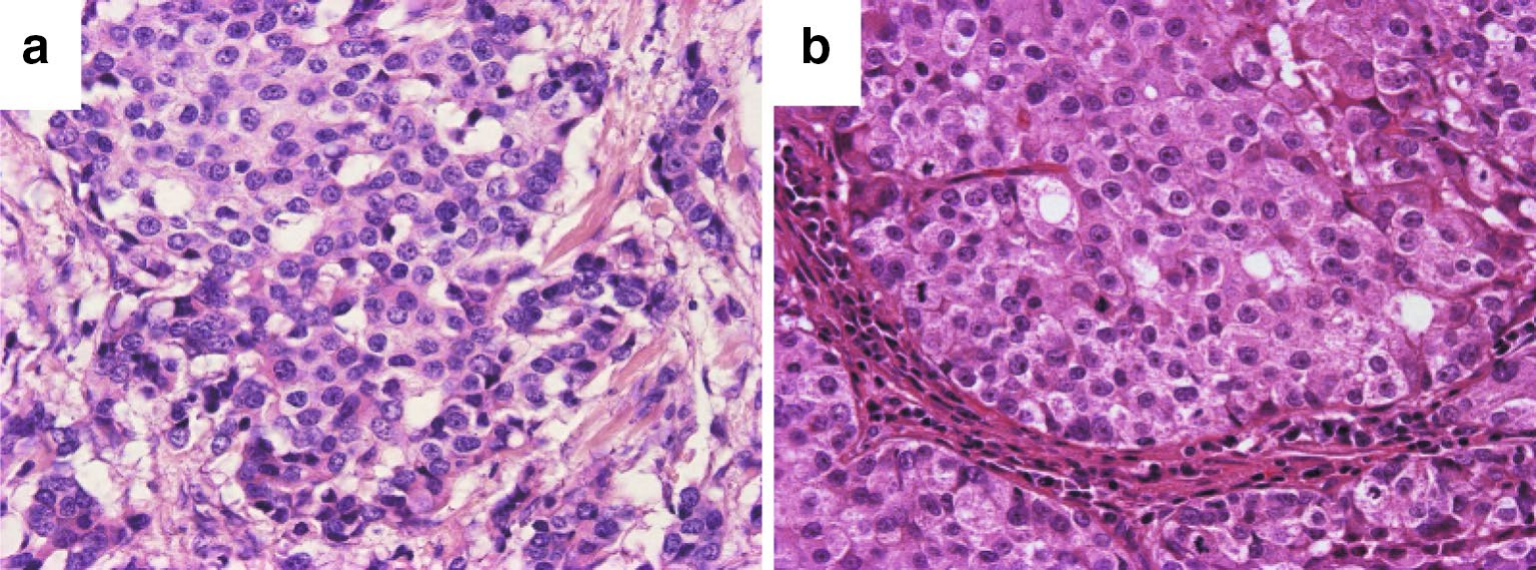
**a** The invasive ductal breast carcinoma specimen that was resected when the patient was 32 years old (H&E). **b** The lung metastasis from breast cancer that was resected when the patient was 42 years old (H&E)

A 56-year-old woman had undergone a mastectomy with axillary lymph node dissection at 32 years of age (T2N1M0). She received tamoxifen for 2 years after surgery. When she was 42 years old, she was diagnosed with lung metastasis from the breast cancer, and a right upper lung lobe resection and lymph node dissection were performed. Images of the invasive ductal breast carcinoma that was resected when the patient was 32 years old and the lung metastasis from the breast cancer that was resected when the patient was 42 years old are shown in Fig. 1a and b, respectively.

**Fig. 2:**
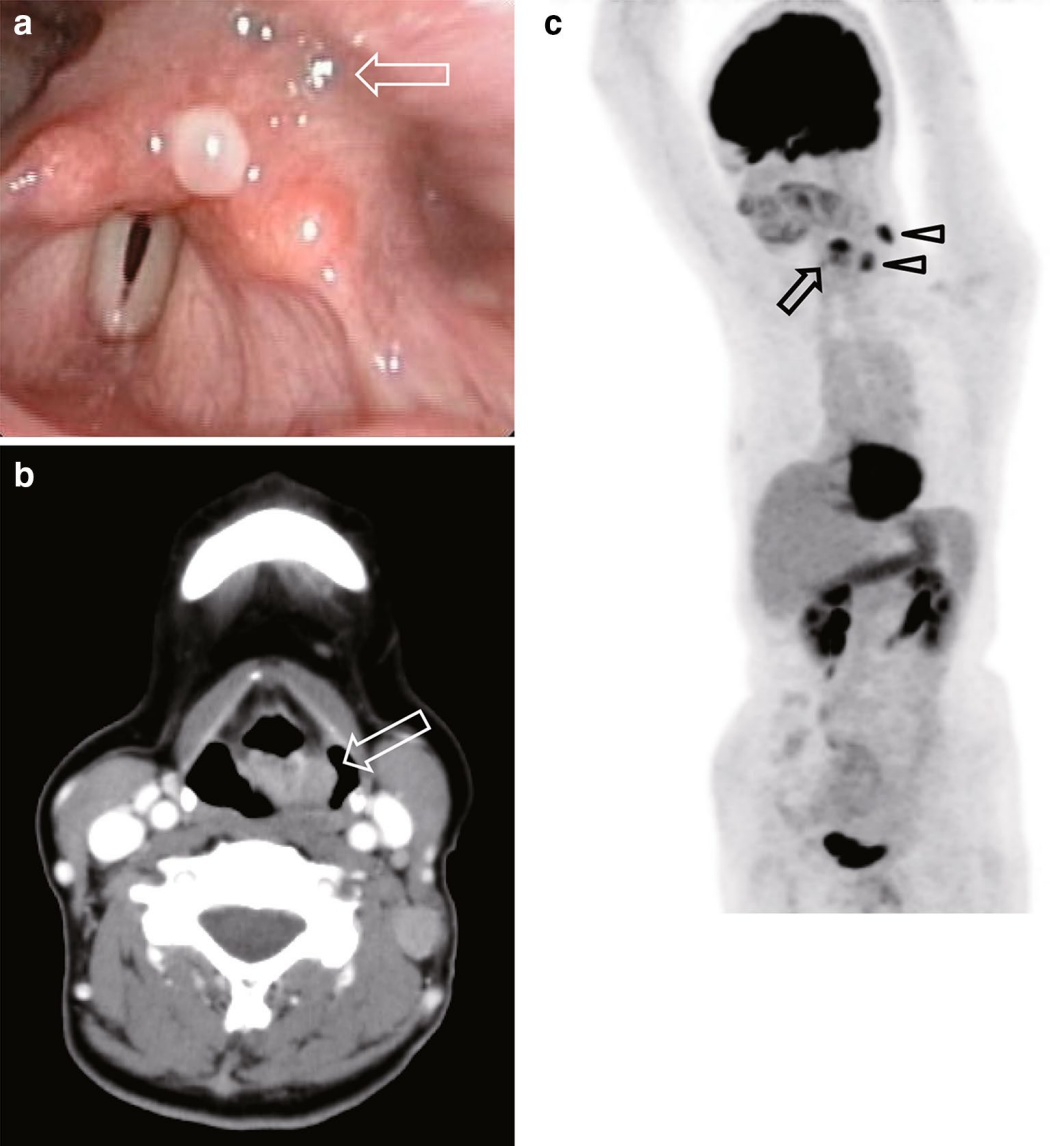
**a** On a hypopharyngeal fiberscopic image, a tumor was observed in the postcricoid area (a *white arrow*). **b** On a contrast-enhanced computed tomography scan of the neck before treatment, an enhanced tumor was observed in the postcricoid area of the hypopharynx (a *white arrow*). **c** On a fluorodeoxyglucose positron emission tomography scan of the neck before treatment, an enhanced tumor was observed in the hypopharynx (a *black arrow*) and the left side of the neck (*arrowhead*)

More than 10 years after the lung resection, when the patient was 56 years old, she was referred to our hospital after complaining of dyspnea on exertion. She had a 3-month history of weight loss and a left neck mass. On hypopharyngoscopy, a tumor lesion was found in the left anterior wall of the hypopharynx, and the patient experienced bilateral vocal cord paralysis (Fig. 2a). A contrast-enhanced computed tomography (CT) scan of the neck revealed a 24-mm heterogeneously enhanced tumor that extended through the left anterior wall of the hypopharynx (Fig. 2b). We performed a tracheostomy, hypopharyngeal biopsy, and lymph node extraction. Moreover, fluorodeoxyglucose positron emission tomography revealed high-level accumulation in the primary tumor, with a maximum standardized uptake value of 5.6, and metastatic neck lymph nodes. There was no evidence of a primary lung tumor or other distant metastases (Fig. 2c).

**Fig. 3:**
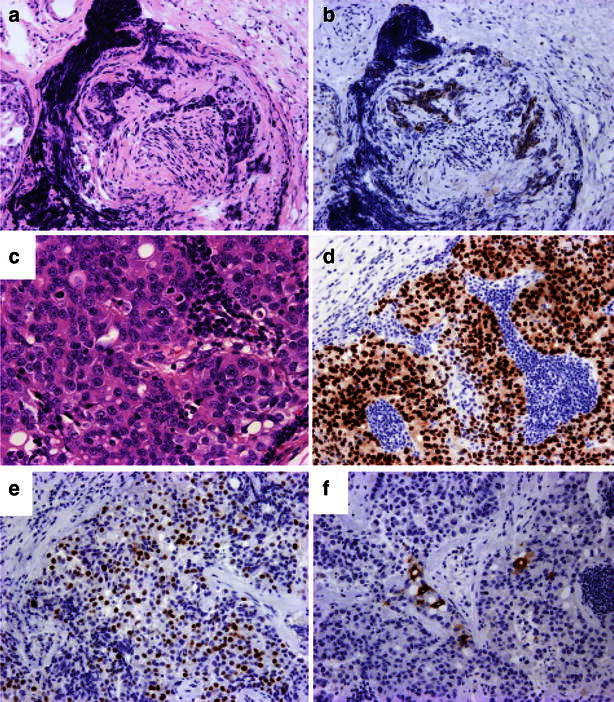
Hematoxylin-eosin staining (H&E) and immunohistochemical studies. **a** Microscopic photos of the hypopharyngeal biopsy specimen (H&E). **b** The hypopharyngeal biopsy sample was diagnosed as metastatic breast carcinoma after positive ER immunostaining. **c** Microscopic photos of the lymph node specimen (H&E). **d** Positive ER immunostaining. **e** Positive progesterone receptor immunostaining. **f** Positive mammaglobin immunostaining

A pathologist analyzed the hypopharyngeal biopsy sample (Fig. 3a, b) and diagnosed the neck lymph node lesion as a hypopharyngeal metastasis from breast cancer. The biopsy sample was estrogen receptor (ER)-positive, progesterone receptor (PR)-positive, and human epidermal growth factor receptor type 2 (HER2)-negative (Fig. 3c–f). The patient completed oral aromatase inhibitor therapy and has remained alive after laryngeal preservation and hypopharyngeal tumor resection for the past 3 years. There was no evidence of any other active disease. The patient responded well, but she showed no objective improvement in laryngeal nerve function with a tracheostomy tube. This patient provided written informed consent under a protocol approved by the Institutional Review Boards at the Hamamatsu University School of Medicine.

## E-cadherin, VEGF-A, and VEGFR2 expression and methylation analyses

**Fig. 4:**
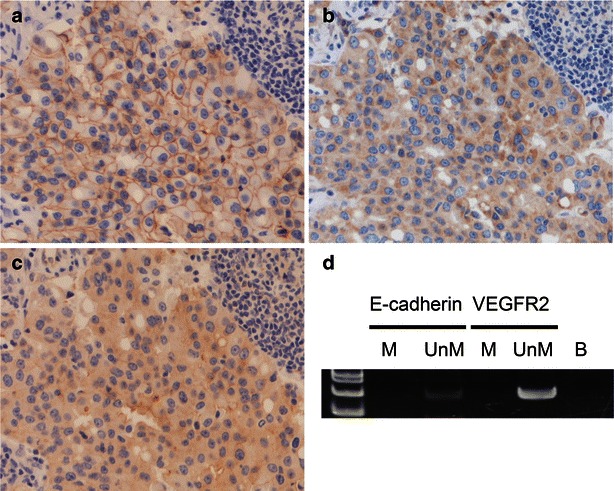
**a** E-cadherin was expressed in the lymph node specimen. **b** Positive VEGF-A receptor immunostaining. **c** Positive VEGFR2 receptor immunostaining. **d** Electrophoresis of methylation-specific PCR products that were amplified using DNA from the lymph node specimen. The results show that only unmethylated alleles of *CDH1* and *VEGFR2* were detected. *M* methylated alleles, *UnM* unmethylated alleles, *B* water blank

**Fig. 5:**
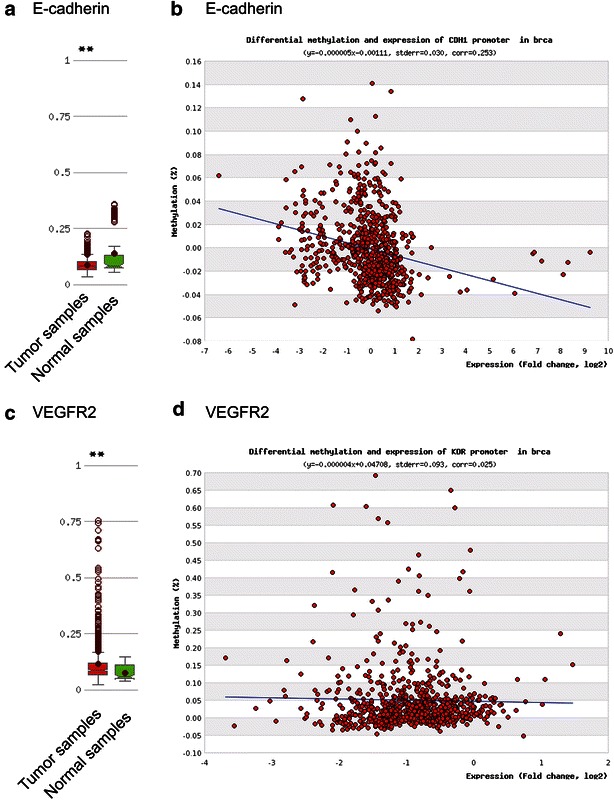
DNA methylation and expression data from the TCGA database of invasive breast carcinoma. **a** Distribution of *E*-*cadherin* DNA methylation between breast cancer samples and normal samples (P < 0.005). **b** A representative result showing the inverse correlation between DNA methylation at the *E*-*cadherin* CpG site and *E*-*cadherin* expression in breast cancer. The Spearman rank correlation coefficient (corr) is shown. **c** Comparison of *VEGFR2* gene expression in tumor samples and matched normal samples (P < 0.005). **d** The relationship between DNA methylation and mRNA expression of the *VEGFR2* gene

We sought to determine a mechanism leading to metastasis by focusing on E-cadherin, vascular endothelial growth factor A (VEGF-A), and vascular endothelial growth factor receptor 2 (VEGFR2). We examined E-cadherin, VEGF-A, and VEGFR2 protein expression levels. On immunohistochemical analysis, the neck lymph node lesion strongly expressed E-cadherin (Fig. 4a), VEGF-A (Fig. 4b), and VEGFR2 (Fig. 4c). Genomic DNA was extracted from the neck lymph nodes using the QIAamp DNA FFPE Tissue Kit (QIAGEN, Hilden, Germany). Bisulfite modification of genomic DNA converts unmethylated cytidine residues to uradine residues that are then converted to thymidine residues during subsequent polymerase chain reaction (PCR) (Misawa et al. 2015). The primers listed in Table 1 were used to analyze *E*-*cadherin* and *VEGFR2* methylation statuses. The PCR products were separated by electrophoresis through a 9 % polyacrylamide gel and stained with ethidium bromide. For *E*-*cadherin* and *VEGFR2*, only unmethylated alleles were detected (Fig. 4c). Neck lymph node tumors without *E*-*cadherin* and *VEGFR2* promoter methylation exhibited relatively robust protein expression levels. DNA methylation and mRNA expression data for invasive breast carcinomas were collected from MethHC: a database of DNA methylation and gene expression in human cancer (http://methhc.mbc.nctu.edu.tw/php/index.php) in April 2016 (Fig. 5) (Huang et al. 2015).

**Table 1:** Primer list of methylation analysis

MSP/UMSP	Gene	Forward/reverse	Sequence
MSP	E-cadherin	Forward	TTAGGTTAGAGGGTTATCGCGT
		Reverse	TAACTAAAAATTCACCTACCGAC
UMSP	E-cadherin	Forward	TAATTTTAGGTTAGAGGGTTATTGT
		Reverse	CACAACCAATCAACAACACA
MSP	VEGFR2	Forward	TCGAGTTTTGGGTATTTCGTTCGGT
		Reverse	AACGACCCGAATCTCCACGCA
UMSP	VEGFR2	Forward	TTGAGTTTTGGGTATTTTGTTTGGT
		Reverse	AACAACCCAAATCTCCACACA

## Discussion

Metastatic breast cancer causes symptoms that differ based on the location of the metastasis (Weng et al. 2014). This patient was referred to our hospital after complaining of dyspnea upon exertion. The metastatic breast cancer was difficult to diagnose from the observation of the mucosal hypopharyngeal surface. To ascertain distant metastatic breast carcinoma, immunohistochemistry should be performed to detect specific mark-ers (Kamby et al. 1988) (Weng et al. 2014). At least two types of markers must be evaluated: markers that are expressed similarly in the original and metastatic lesions and markers that can be used to differentiate between metastatic lesions and surrounding components (Zhang et al. 2013).

Approximately 20–40 % of patients with ER-positive breast cancer eventually develop recurrences in distant organs, and half of these events occur at least 5 years after diagnosis of the primary tumor. This phenomenon is especially pronounced in patients with ER-positive breast cancer, suggesting that E-positive cancer cells may remain dormant for a protracted period, despite adjuvant therapy (Zhang et al. 2013). Late recurrence is thought to be to the result of cancerous cells becoming activated from a dormant state, in which little or no de novo DNA transcription occurs and minimal protein translation from RNA occurs only to maintain the vegetative functions that sustain cell viability (Meltzer 1990). These findings suggest that the presence of certain cellular receptors may correlate with biological behavior of tumors, as manifested by differences in response to therapy and metastatic distributions.

Many human metastatic breast cancer lesions express membranous E-cadherin, whereas their paired primary tumors are E-cadherin-negative (Chao et al. 2010). Although E-cadherin re-expression and accompanying morphological changes have been achieved, any subsequent full or partial mesenchymal to epithelial transition has not been adequately assessed (Chao et al. 2012). E-cadherin re-expression due to loss of methylation suggests a functional mechanism by which the microenvironment modulates the mesenchymal to epithelial phenotypic switch (Taylor et al. 2014; Wendt et al. 2011).

VEGF-A and VEGFR2 are often co-expressed in breast cancer and potentially affect cellular pathways and the expression levels of key proteins that are targeted by endocrine therapy, such as ER (Mele et al. 2010). Expression of tumor-specific VEGFR2 is a predictive marker for response to tamoxifen in breast cancer patients (Ryden et al. 2005a). Elevated VEGF-A and VEGFR2 expression levels are associated with poor prognosis and poor response to tamoxifen therapy, suggesting that the combination of anti-hormone treatment with an anti-angiogenic strategy should be tested in clinical trials (Patel et al. 2010; Ryden et al. 2005b).

Patients with ER-positive breast cancers generally have a more favorable clinical outcomes, better prognoses, and better patterns of recurrence. Anti-estrogens, such as tamoxifen, and aromatase inhibitors, such as letrozole, can effectively control the disease and induce tumor responses in a large proportion of patients (Milani et al. 2014). For many years, tamoxifen was the most widely used first-line hormonal therapy for post-menopausal patients with hormone-sensitive advanced or metastatic breast cancer. Aromatase inhibitors, which have shown superior efficacy in advanced disease when compared with tamoxifen, have now largely replaced tamoxifen as first-line therapy in postmenopausal women (Riemsma et al. 2010).

## Conclusions

In conclusion, hypopharyngeal metastasis of breast cancer is extremely rare. This case highlights the importance of immunohistochemical analysis in hypopharyngeal tumor diagnosis. ER, VEGF, and VEGFR2 expression levels are used when prescribing aromatase inhibitors as adjuvant treatment for postmenopausal patients. The patient in this study is currently alive after laryngeal preservation and hypopharyngeal tumor resection 3 years ago. E-cadherin and VEGFR2 expression levels and methylation statuses may be used to improve our understanding of ER-positive breast cancer reactivation after dormancy. However, these associations should be interpreted with caution because we have examined only one case.
